# Supplementary material for: Size Matters in the Cytotoxicity of Polydopamine Nanoparticles in Different Types of Tumors
Source: Cancers (Basel). 2019 Oct 29;11(11):1679. doi: 10.3390/cancers11111679 (PMC6896006; doi:10.3390/cancers11111679)
Supplement: Supplementary file 1 [file cancers-11-01679-s001.pdf]

# Supplementary Materials: Size Matters in the Cytotoxicity of Polydopamine Nanoparticles in Different Types of Tumors

**Celia Nieto, Milena A. Vega, Jesús Enrique, Gema Marcelo and Eva M. Martín del Valle**

**a** Breast carcinoma - BT474 cell line

| | 115 nm | | | 200 nm | | |
|---|---|---|---|---|---|---|
| [PD NPs] (mg/mL) | 24h | 48h | 72h | 24h | 48h | 72h |
| 0.0074 | 88.6 ± 1.7 | 81.0 ± 1.3 | 77.2 ± 3.2 | 92.5 ± 2.5 | 86.4 ± 1 | 82.7 ± 1.2 |
| 0.015 | 82.6 ± 3 | 78.3 ± 2.8 | 71.8 ± 2.3 | 86.5 ± 0.2 | 83.9 ± 0.5 | 79.1 ± 1.6 |
| 0.029 | 77.4 ± 2.1 | 75.7 ± 1.5 | 65.2 ± 3.8 | 80.5 ± 0.8 | 73.2 ± 1 | 71.1 ± 0.8 |
| 0.042 | 69.0 ± 0.7 | 64.9 ± 1.4 | 58.8 ± 0.7 | 75.4 ± 1.4 | 69.3 ± 0.5 | 64.7 ± 0.8 |

**b** Colon carcinoma – HTC116 cell line

| | 115 nm | | | 200 nm | | |
|---|---|---|---|---|---|---|
| [PD NPs] (mg/mL) | 24h | 48h | 72h | 24h | 48h | 72h |
| 0.0074 | 93.4 ± 0.6 | 88.5 ± 1.4 | 85.9 ± 1.3 | 92.4 ± 3 | 89.1 ± 4 | 87.0 ± 3 |
| 0.015 | 84.1 ± 1.2 | 81.9 ± 1.1 | 77.9 ± 1.5 | 88.4 ± 4 | 82.2 ± 1 | 77.5 ± 1 |
| 0.029 | 79.9 ± 0.5 | 73.8 ± 1.3 | 69.0 ± 1.3 | 84.6 ± 3 | 79.5 ± 2 | 72.8 ± 0.5 |
| 0.042 | 70.7 ± 2.3 | 66.5 ± 1.1 | 63.1 ± 2.4 | 78.2 ± 4 | 75.9 ± 3 | 70.3 ± 2 |

**c** Liver carcinoma – HEPG2 cell line

| | 115 nm | | | 200 nm | | |
|---|---|---|---|---|---|---|
| [PD NPs] (mg/mL) | 24h | 48h | 72h | 24h | 48h | 72h |
| 0.0074 | 93.1 ± 1.4 | 86.9 ± 1.6 | 83.5 ± 1.2 | 94.9 ± 2 | 89.4 ± 0.7 | 86.5 ± 3 |
| 0.015 | 81.3 ± 0.5 | 78.4 ± 1.4 | 73.5 ± 1.4 | 89.8 ± 3 | 83.3 ± 0.8 | 80.9 ± 3 |
| 0.029 | 74.7 ± 0.6 | 68.8 ± 0.7 | 65.9 ± 1 | 85.7 ± 1 | 81.1 ± 0.8 | 74.7 ± 2 |
| 0.042 | 68.9 ± 1.3 | 63.3 ± 1.5 | 60.1 ± 0.8 | 79.1 ± 1.5 | 75.8 ± 3 | 67.4 ± 1 |

**d** Lung carcinoma – H460 cell line

| | 115 nm | | | 200 nm | | |
|---|---|---|---|---|---|---|
| [PD NPs] (mg/mL) | 24h | 48h | 72h | 24h | 48h | 72h |
| 0.0074 | 91.8 ± 0.9 | 88.2 ± 2.6 | 85.9 ± 3.4 | 94.2 ± 1.5 | 89.8 ± 0.5 | 87.4 ± 2.5 |
| 0.015 | 87.2 ± 1.3 | 82.3 ± 2.2 | 80.0 ± 4.5 | 92.0 ± 1.5 | 84.2 ± 0.8 | 82.7 ± 0.8 |
| 0.029 | 86.5 ± 1.2 | 79.0 ± 3.4 | 77.7 ± 1.5 | 89.0 ± 1.3 | 80.0 ± 1 | 77.3 ± 2.5 |
| 0.042 | 84.3 ± 1.2 | 75.3 ± 1.8 | 74.3 ± 2.9 | 85.3 ± 2 | 78.9 ± 2.5 | 76.6 ± 2 |

**e** Stromal cells – HS5 cell line

| | 115 nm | | | 200 nm | | |
|---|---|---|---|---|---|---|
| [PD NPs] (mg/mL) | 24h | 48h | 72h | 24h | 48h | 72h |
| 0.0074 | 96.7 ± 0.9 | 93.9 ± 1.2 | 93.3 ± 0.4 | 97.9 ± 1.5 | 97.3 ± 3 | 94.5 ± 1 |
| 0.015 | 93.6 ± 0.4 | 89.8 ± 1.7 | 85.9 ± 0.8 | 97.3 ± 3 | 88.0 ± 3 | 85.0 ± 0.8 |
| 0.029 | 90.0 ± 1.1 | 86.6 ± 1.2 | 83.9 ± 2.3 | 95.1 ± 4.5 | 86.2 ± 1.5 | 84.3 ± 1.5 |
| 0.042 | 83.2 ± 0.5 | 79.5 ± 0.5 | 75.4 ± 1.1 | 94.6 ± 1.8 | 79.6 ± 2 | 76.3 ± 2 |

**Figure S1.** Data corresponding to MTT results shown in Figure 2 with the BT474 (**a**), HTC116 (**b**), HEPG2 (**c**), H460 (**d**) and HS5 (**e**) cell lines. Shown results are the average cellular viability percentage ± SD of three replicas for each treatment.

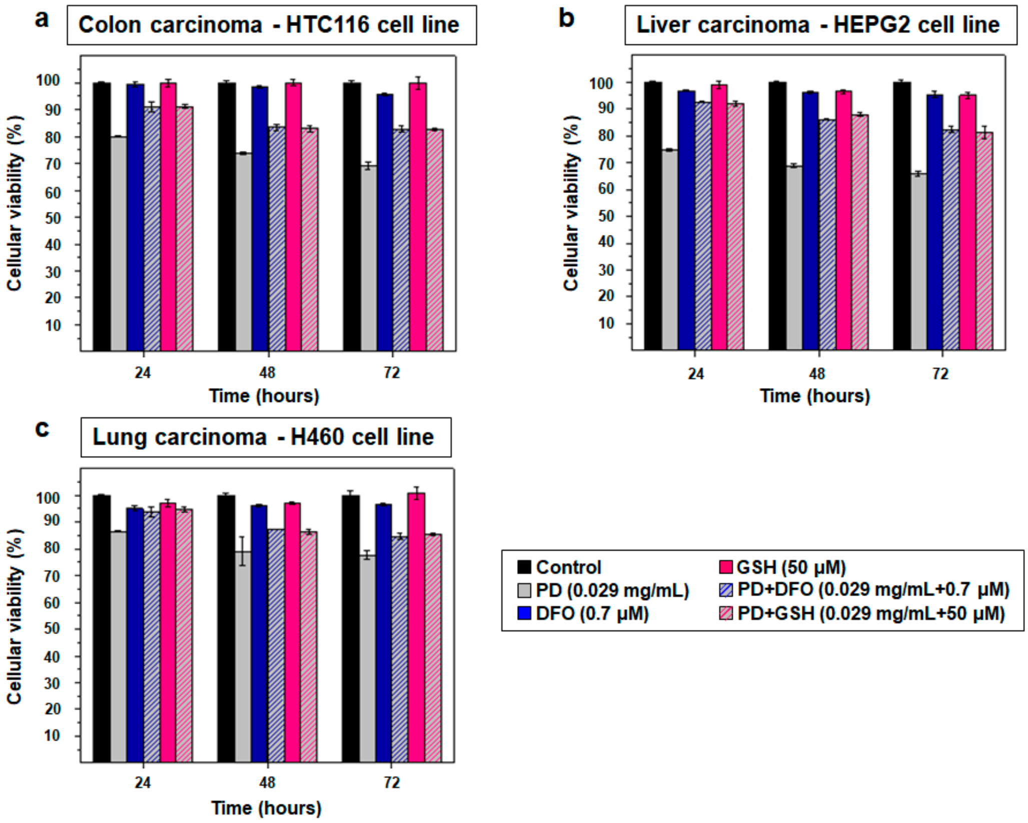


**Figure S2.** Results of the MTT assays carried out with HTC116 (**a**), HEPG2 (**b**) and H460 (**c**) cells after a co-treatment of 115 nm PD NPs (0.029 mg/mL, blue) with DFO (0.7 µM, green) and GSH (50 µM, pink). Shown results are again the mean ± SD of three replicas for each treatment.

© 2019 by the authors. Licensee MDPI, Basel, Switzerland. This article is an open access article distributed under the terms and conditions of the Creative Commons Attribution (CC BY) license (http://creativecommons.org/licenses/by/4.0/).
